# Scintigraphic Imaging of Neovascularization With ^99m^Tc-3PRGD_2_ for Evaluating Early Response to Endostar Involved Therapies on Pancreatic Cancer Xenografts *In Vivo*

**DOI:** 10.3389/fonc.2021.792431

**Published:** 2021-12-15

**Authors:** Xiaona Jin, Chengyan Dong, Kun Zheng, Ximin Shi, Yu Liu, Li Huo, Fan Wang, Fang Li

**Affiliations:** ^1^ Department of Nuclear Medicine, Peking Union Medical College Hospital, Chinese Academy of Medical Sciences and Peking Union Medical College, Beijing, China; ^2^ Beijing Key Laboratory of Molecular Targeted Diagnosis and Therapy in Nuclear Medicine, Beijing, China; ^3^ GE Healthcare China, Beijing, China; ^4^ Medical Isotopes Research Center, Peking University, Beijing, China

**Keywords:** Endostar, ^99m^Tc-3PRGD_2_, antiangiogenesis, scintigraphic imaging, microvessel density

## Abstract

**Background:**

Molecular imaging targeting angiogenesis can specifically monitor the early therapeutic effect of antiangiogenesis therapy. We explore the predictive values of an integrin αvβ3-targeted tracer, ^99m^Tc-PEG_4_-E[PEG_4_-c(RGDfK)]_2_ (^99m^Tc-3PRGD_2_), for monitoring the efficacy of Endostar antiangiogenic therapy and chemotherapy in animal models.

**Methods:**

The pancreatic cancer xenograft mice were randomly divided into four groups, with seven animals in each group and treated in different groups with 10 mg/kg/day of Endostar, 10 mg/kg/day of gemcitabine, 10 mg/kg/day of Endostar +10 mg/kg/day of gemcitabine at the same time, and the control group with 0.9% saline (0.1 ml/day). ^99m^Tc-3PRGD_2_ scintigraphic imaging was carried out to monitor therapeutic effects. Microvessel density (MVD) was measured using immunohistochemical staining of the tumor tissues. The region of interest (ROI) of tumor (T) and contralateral corresponding site (NT) was delineated, and the ratio of radioactivity (T/NT) was calculated. Two-way repeated-measure analysis of variance (ANOVA) was used to assess differences between treatment groups.

**Results:**

Tumor growth was significantly lower in treatment groups than that in the control group (p < 0.05), and the differences were noted on day 28 posttreatment. The differences of ^99m^Tc-3PRGD_2_ uptakes were observed between the control group and Endostar group (p = 0.033) and the combined treatment group (p < 0.01) on day 7 posttreatment and on day 14 posttreatment between the control group and gemcitabine group (p < 0.01). The accumulation of ^99m^Tc-3PRGD_2_ was significantly correlated with MVD (r = 0.998, p = 0.002).

**Conclusion:**

With ^99m^Tc-3PRGD_2_ scintigraphic imaging, the tumor response to antiangiogenic therapy, chemotherapy, and the combined treatment can be observed at an early stage of the treatments, much earlier than the tumor volume change. It provides new opportunities for developing individualized therapies and dose optimization.

## Introduction

Inhibition of angiogenesis causes vascular degeneration, hinders the delivery of oxygen and nutrients, and eventually leads to tumor hunger. Antiangiogenic therapy has been approved as an effective strategy to inhibit tumor growth and affect metastatic spread in many countries, providing a novel treatment approach for cancer patients (1, 2). As a recombinant human endostatin, Endostar is mainly used for cancer treatment as an antiangiogenic agent (3, 4). It was approved by the China Food and Drug Administration (CFDA) for lung cancer treatment in 2005. Endostar has been used in the treatment of a variety of cancers for antiangiogenesis effect, including non-small cell lung cancer, breast cancer, melanoma, and gastric cancer (5–10). Nevertheless, the benefits of Endostar on pancreatic cancer are currently poorly known. Endostar was effective in the treatment of advanced pancreatic neuroendocrine tumors combined with temozolomide or dacarbazine + 5-FU, and the combinations were well tolerated (11). Antiangiogenic therapeutics or inhibitors of proangiogenic kinase pathways could antagonize the growth-promoting effect of cantharidin and present additive antitumor effects, exhibiting adequate efficacy. Endostar has shown a good safety profile and tolerance in previous studies, without common toxicity of other VEGF or VEGFR inhibitors, such as hypertension and proteinuria (6, 12–15).

In the past years, clinical trials of antiangiogenic therapy with anti-VEGF (bevacizumab) or anti-VEGFR (sorafenib, axitinib) for pancreatic cancer have been carried out (16–20). As often observed in clinical trials, only some patients benefit from treatment (21, 22). Therefore, there is an urgent need to develop an alternative approach to select patients who will benefit from antiangiogenic therapies, detect emerging drug resistance, and monitor early treatment outcomes (23).

A histopathologic evaluation of microvessel density (MVD) has been suggested as a prognostic indicator of progression, but it is not suitable for repeated evaluation of tumor angiogenesis because of the invasive nature of the procedure (24). Noninvasive imaging techniques such as dynamic contrast-enhanced (DCE) magnetic resonance imaging (MRI) or computed tomography (CT) can evaluate tumor blood flow and volume but have limited capability to quantify the changes of tumor vessels after treatment (25–27). Positron emission tomography (PET) has been used to monitor antiangiogenic therapy by measuring glucose metabolism changes with approp^18^F-FDG (2-deoxy-2-18F-fluoro-D-glucose), but ^18^F-FDG may not be an appropriate modality as a non-specific tracer. Therefore, molecular imaging targeting specific pathways related to angiogenesis is necessary to specifically monitor some molecular sequence. As an early treatment effect, its advantage is to allow repeated non-invasive follow-ups in the treatment process (28, 29).

Integrin αvβ3 imaging may provide a new method for evaluating tumor angiogenesis and monitoring the response to antiangiogenic therapy (28). The Arg–Gly–Asp (RGD) sequence was known to be associated with integrins expressed on the surface of angiogenic vessels or tumor cells αvβ3 (30). Thus, various radiolabeled derivatives of RGD peptides have been developed for angiogenesis imaging by PET imaging, such as ^18^F-FPRGD_2_ and ^68^Ga-NOTA-PRGD_2_, and single-photon emission computed tomography (SPECT) imaging for the diagnosis of cancers, such as ^99m^Tc-3PRGD_2_ (31–35). RGD-based PET tracers have been evaluated to be comparable to ^18^F-FDG for lesion detection in clinical studies, with high specificity and long tumor retention. Especially for gliomas and brain metastases, PET imaging with RGD analogues showed a much higher tumor-to-background than ^18^F-FDG did (36–38). ^68^Ga-3PRGD_2_ PET reflected the tumor response to Endostar antiangiogenic therapy much earlier and more accurately than did ^18^F-FDG metabolic imaging (31–35). Compared with the tracers for PET, ^99m^Tc-3PRGD_2_ is a SPECT tracer with wider availability, especially in underdeveloped areas. Because of its simple, efficient, and repeatable preparation procedure, ^99m^Tc-3PRGD_2_ is easy for routine clinical use (39, 40).

We tried to evaluate the value of ^99m^Tc-3PRGD_2_ as a binding integrin α_v_β_3_ imaging agent in monitoring the efficacy of Endostar antiangiogenesis therapy and chemotherapy in animal models, to find a specific way for early monitoring the therapeutic effects and evaluating the follow-ups during the whole therapy. In this study, we also involved gemcitabine, the standard of care for the first-line treatment of metastatic pancreatic cancer globally, to further evaluate the capability of ^99m^Tc-3PRGD_2_ in treatment monitoring (39, 40).

## Method

### Radiopharmaceutical Preparation

Synthesis of the labeling precursor, kit preparation, and subsequent ^99m^Tc-labeling were performed as previously described (35). Briefly, the kit for the preparation of ^99m^Tc-3PRGD_2_ was formulated by combining 20 mg of hydrazinonicotinamide-3PRGD_2_, 5 mg of trisodium triphenylphosphine-3,39,399-trisulfonate (TPPTS), 6.5 mg of tricine, 40 mg of mannitol, 38.5 mg of disodium succinate hexahydrate, and 12.7 mg of succinic acid. For ^99m^Tc radiolabeling, to the kit vial was added 1 ml of 1.110–1.850 MBq (30–50 mCi) of $99^{m}\text{Tc}{\text{O}}_{4}^{-}$ saline solution, and then the vial was water-bathed at 100°C for 15–20 min [MS data shown in previous article ref. (33)]. The resulting solution was analyzed by instant thin-layer chromatography using Gelman Sciences silica-gel paper strips and a 1:1 mixture of acetone and saline as eluant. The radiochemical purity was always greater than 95%. The reaction mixture was then diluted to approximately 370 MBq/ml (10 mCi/ml) with saline and was filtered with a 0.20-mm Millex-LG filter (EMD Millipore). Each animal was injected with 7.4–11.1 MBq (0.2–0.3 mCi) of ^99m^Tc-3PRGD_2_ per mouse.

### Animal Model Establishment

Female BALB/c mice (5 weeks of age) were purchased from Vital River Lab Animal Technology Co., Ltd. The PANC-1 mouse model was established by subcutaneous injection of 2 × 10^6^ PANC-1 cells into the right shoulders of mice. Once the tumor diameter reached 5–7 mm, the mice were initiated with treatment (~2 weeks after inoculation of PANC-1 cells).

### Treatment Protocols

**Figure 1:**
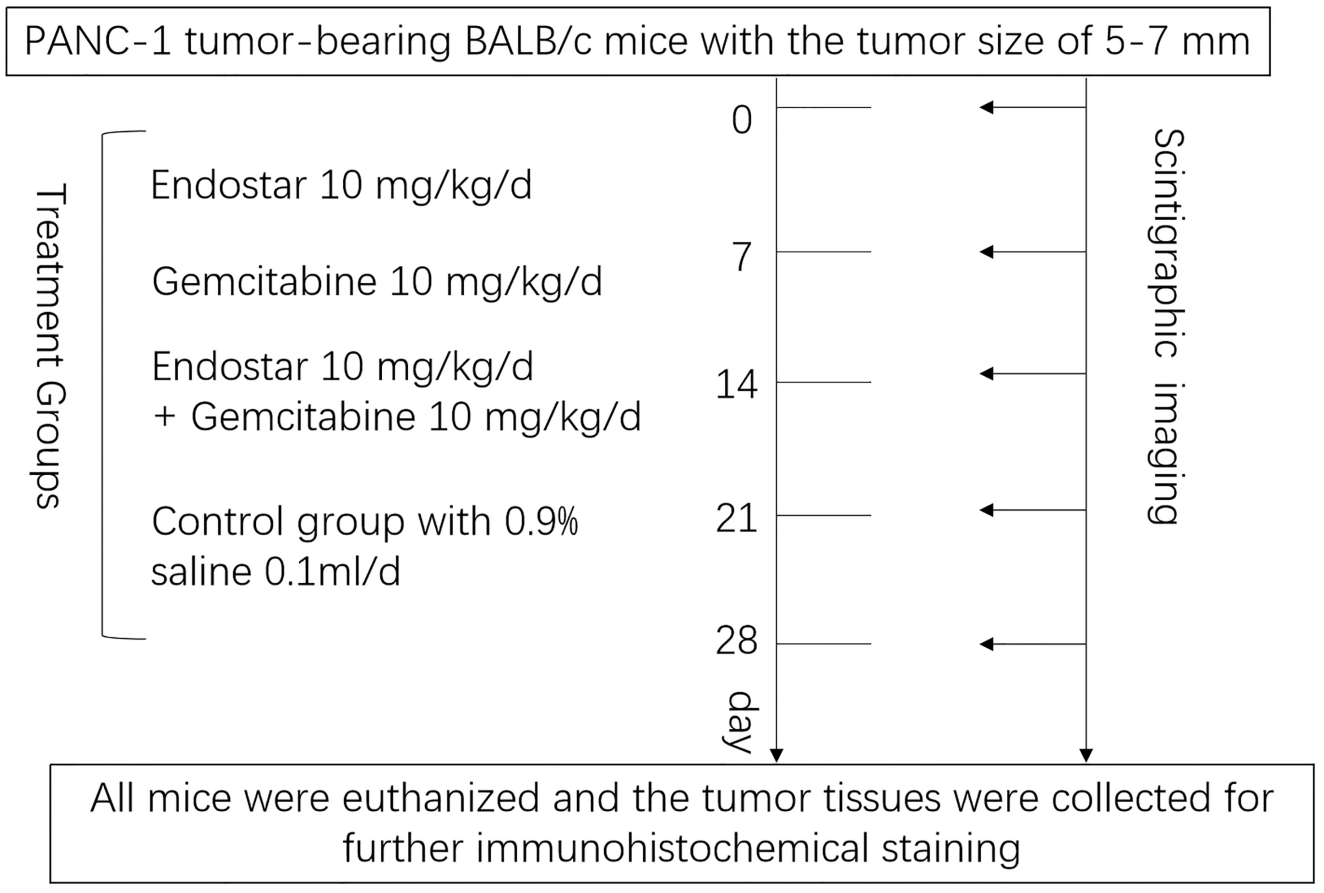
Therapy and imaging protocols.

The study flowchart is given in **Figure 1**. PANC-1 tumor-bearing BALB/c mice with a tumor size of 5–7 mm were randomly divided into four groups (n = 7 mice per group). The first group was intraperitoneally injected with 10 mg/kg Endostar, the second group was intraperitoneally injected with 10 mg/kg gemcitabine, the third group was intraperitoneally injected with 10 mg/kg Endostar, and 10 mg/kg gemcitabine at the same time and 0.9% saline were used as the negative control. The treatments were performed daily for 28 days continuously. The tumor size was measured daily with a digital caliper, and the formula (volume = 1/2 length × width× width) was used to calculate the tumor volume. Body weight was monitored daily to assess potential toxicity. All mice were euthanized, and the tumor tissues were harvested for further immunohistochemical staining when the treatment was complete.

### Imaging Protocol

The scanner was a dual-head γ-camera (Siemens e.cam, Germany), using low-energy high-resolution collimators and a 20% energy window centered on 140 keV. Static planar scans of the mice were obtained at 1.5 h postinjection (p.i.) under isoflurane anesthesia. The acquisition count was 3 × 10^5^. The matrix is 256 × 256, and the magnification is 1.33. The regions of interest (ROIs) of the tumor (T) and non-target (NT, contralateral muscles) were delineated, and the ratios of radioactivity (T/NT) were calculated. The study flowchart is given in **Figure 1**.

### Immunohistochemical Studies

Using formalin-fixed paraffin-embedded tissue sections and the envision method, CD31-stained slides were examined under appropriate pretreatment to determine MVD in tumor tissue samples. Pathologists selected representative specimens according to the quality and quantity of embedded tissues. On the CD31-stained slides, microvessel density was counted in three fields at a magnification of ×200. Microvessel counts were considered to be all round, oval, or irregular structures with positive staining, which were separated from other contour or connective tissue elements. Vessels with muscularis and necrotic areas were excluded.

### Statistical Analysis

Quantitative and semiquantitative data were expressed as the mean ± SD and analyzed using SPSS version 17.0 (IBM, Chicago, IL, USA). Mean values were compared using one-way analysis of variance (ANOVA) or Student’s t-test. Two-way repeated-measure analysis of variance (ANOVA) was used to evaluate the differences between different treatment groups.

## Results

### Effect of Treatments on Tumor Growth

**Figure 2:**
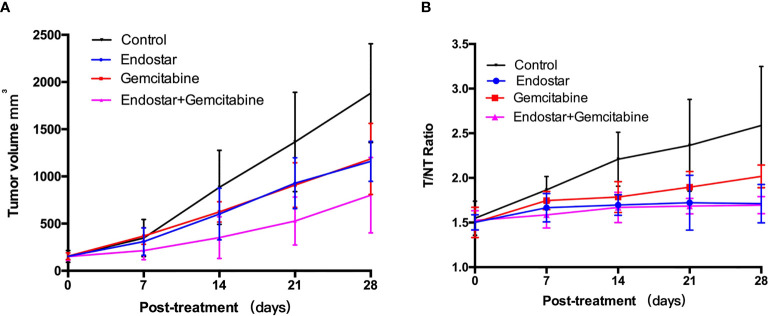
**(A)** Tumor growth profiles of the control group and treatment groups (7 mice per group). PANC-1 tumor-bearing mice were treated *via* intraperitoneal injection. Saline-treated animals served as controls. **(B)** ^99m^Tc-3PRGD_2_ tumor uptake (T/N) in each groups.

There was no significant tumor growth inhibition observed in the Endostar or gemcitabine group before day 21 posttreatment, compared with the control group (p > 0.05). The difference between the Endostar + gemcitabine group and the control group was observed on day 14 posttreatment (p < 0.01). At the end of the treatment (day 28 posttreatment), the tumor growth in the control group was rapid with the tumor sizes reaching over 1,881 ± 523 mm^3^, but 1,160 ± 212 mm^3^ in the Endostar group, 1,171 ± 496 mm^3^ in the gemcitabine group, and 801 ± 399 mm^3^ in the Endostar + gemcitabine group. There were differences between the treatment groups and the control group (p < 0.05, **Table 1**), demonstrating the tumor growth inhibition effect of treatments. Two-way repeated-measure ANOVA was statistically significant for differences between the three treatment groups and the control group (**Table 2** and **Figure 2A**). Tumor growth was significantly faster in the control group than in all other groups (p < 0.05) and was slower in the Endostar + gemcitabine group than the gemcitabine (p = 0.021) and Endostar groups (p = 0.034). Treatment was the only regimen that resulted in slowing of the growth of the tumor volume.

**Table 1:** Unpaired t-test (treatment groups vs. control group).

TimeGroups	Day 0		Day 7		Day 14		Day 21		Day 28	
	Tumor volume	T/NT	Tumor volume	T/NT	Tumor volume	T/NT	Tumor volume	T/NT	Tumor volume	T/NT
Endostar vs. control	p = 0.953	p = 0.573	p = 0.696	p = 0.033	p = 0.143	p<0.01	p = 0.07	p = 0.015	p<0.01	p<0.01
Gemcitabine vs. control	p = 0.917	p = 0.630	p = 0.441	p = 0.108	p = 0.258	p<0.01	p = 0.191	p = 0.041	p = 0.023	p = 0.044
Endostar + gemcitabine vs. control	p = 0.959	p = 0.769	p = 0.136	p<0.01	p<0.01	p<0.01	p<0.01	p<0.01	p<0.001	p<0.01

**Table 2:** Repeated-measure ANOVA.

Groups ANOVA	Tumor volume		T/NT	
	F	p	F	p
Endostar vs. control	5.660	0.035	12.981	0.004
Gemcitabine vs. control	4.899	0.047	6.913	0.022
Endostar + gemcitabine vs. control	11.873	0.005	16.133	0.002
Gemcitabine vs. Endostar + gemcitabine	7.086	0.021	11.838	0.005
Endostar vs. gemcitabine	0.047	0.832	4.955	0.051
Endostar vs. Endostar + gemcitabine	5.735	0.034	0.302	0.593

### Monitoring the Efficacy of Antiangiogenic Therapy by Scintigraphic Imaging

**Figure 3:**
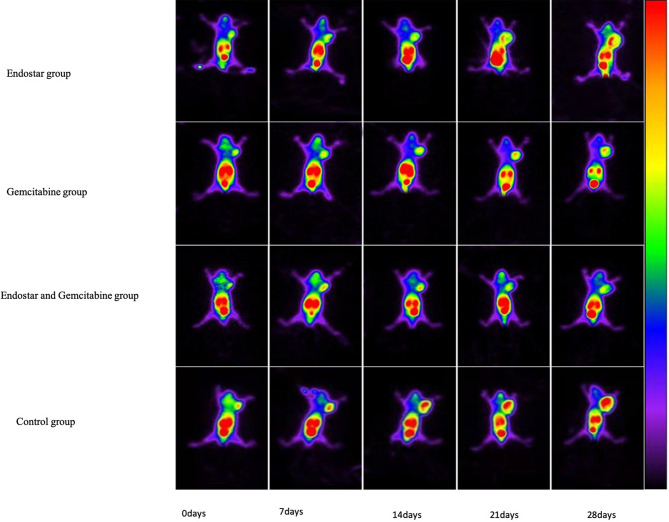
PANC-1 tumor-bearing mice were imaged with ^99m^Tc-3PRGD_2_ SPECT at days 0, 7, 14, 21, and 28 post-treatment in the Endostar group, gemcitabine group, combined therapy group, and control group.

To monitor the effect of antiangiogenic therapy, scintigraphic imaging was performed by using ^99m^Tc-3PRGD_2_ on days 0, 7, 14, 21, and 28 posttreatment (**Figure 3**), respectively. At baseline, the tumor uptake values (T/NT) of ^99m^Tc-3PRGD_2_ were 1.50 ± 0.08 (Endostar group), 1.50 ± 0.17 (gemcitabine group), 1.52 ± 0.11 (Endostar+ gemcitabine group), and 1.55 ± 0.19 (control group), and T/NT in treatment groups at this time had no significant difference compared to the control group. On day 7 posttreatment, T/NT in the Endostar group was significantly lower than that in the control group (1.67 ± 0.16 vs. 1.87 ± 0.15, p = 0.033), and the difference lasted until the end of treatment (**Table 1**). The difference was also observed between the control group and the Endostar + gemcitabine group. Moreover, the difference between the gemcitabine group and the control group was observed on day 14 posttreatment. For the therapeutic effect evaluated by T/NT (**Figure 2B**), two-way repeated-measure ANOVA was statistically significant for differences between the three treatment groups and the control group, shown in **Table 2**. The T/NT rise was significantly faster in the control group than in all other groups (p < 0.05) and was slower in the Endostar + gemcitabine group than in the gemcitabine group (p = 0.005), but there was no difference between the Endostar + gemcitabine group and the Endostar group (p = 0.593).

### Immunohistochemical Findings

**Figure 4:**
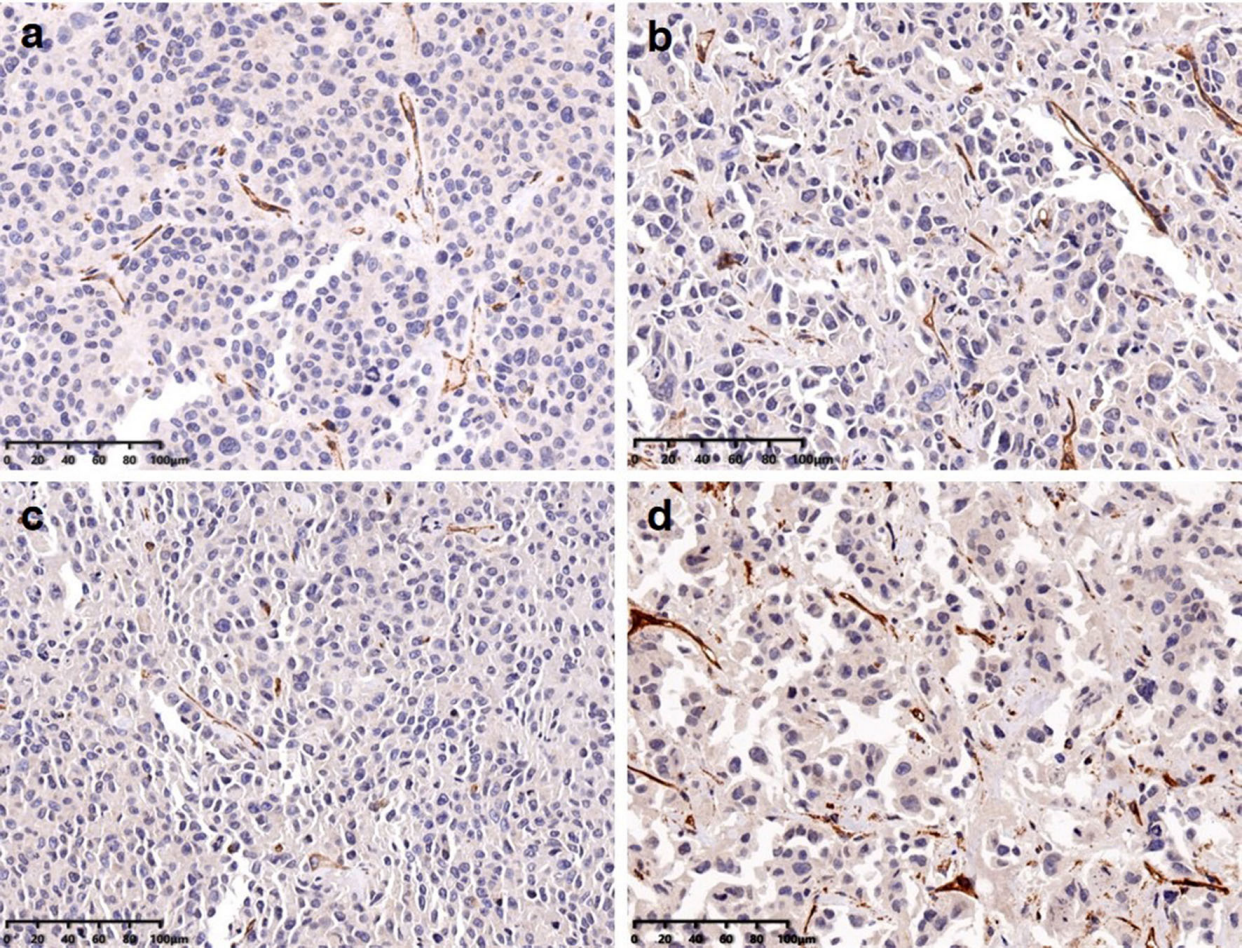
MVD calculated from immunohistochemical stainings in Endostar **(A)**, gemcitabine **(B)**, the combination of those two agents **(C)**, and the control group **(D)**.

Twenty-eight specimens were stained with CD31 to correlate with the imaging findings. The microvessel densities (MVD) were 10.5 ± 1.7, 15.3 ± 2.5, 9.7 ± 1.4, and 23.1 ± 2.7 in the Endostar group, gemcitabine group, Endostar + gemcitabine group, and the control group (**Figure 4**). ^99m^Tc-3PRGD_2_ accumulation was significantly correlated with MVD counted on the CD31-stained slides (r = 0.998, p = 0.002). MVD in the treatment groups was significantly lower than in the control group (p < 0.05). The difference was observed between the Endostar group and the gemcitabine group, but there was no difference between the Endostar group and the Endostar +gemcitabine group.

## Discussion

Pancreatic cancer remains one of the most lethal malignancies, causing a huge incidence rate and mortality worldwide. The 5-year survival rate is 10% in the United States and about 7% in China, because about 80%–85% of patients have unresectable tumors or metastasis at the time of diagnosis (41, 42). The main treatment for patients with advanced disease remains systemic combined chemotherapy (43). Therefore, new effective therapeutic schemes and sensitive evaluation of curative effects by non-invasive imaging methods become highly important. In addition to conventional chemotherapy combinations, multiple trials of antiangiogenic therapy with anti-VEGF (bevacizumab) and anti-VEGFR (sorafenib, axitinib) showed variable results such as positive benefits for patients (44–46) or negative results (15–18, 20). In our study, Endostar was chosen as an antiangiogenic drug for the treatment of pancreatic, alone or combined with gemcitabine. Furthermore, ^99m^Tc-3PRGD_2_ SPECT was used to evaluate the therapeutic effect targeting neovascularization.

The results of the two-way repeated-measure ANOVA showed that all three therapeutic schemes were effective in inhibiting tumor growth. On day 14 posttreatment, the tumor volume of the treated mice in the Endostar + gemcitabine group was significantly smaller than that in the control group, but Endostar or gemcitabine did not induce a significant reduction in the slope of tumor growth, as compared to controls. On day 28 posttreatment, in monotherapy groups, the tumor growth was observed to be lower than that in the control group. Endostar blocks VEGF/VEGFR signaling which hinders tumor growth by regulating the degradation of the existing tumor vascular system and preventing tumor regeneration for a long time (47, 48), while gemcitabine treatment caused cytotoxic damage, abnormal DNA repair, and apoptosis (47, 48). The combination of the two drugs seems to be more effective because of the synergy of the two drugs in two different ways to promote tumor growth.

At the end of the second week, a reduction in ^99m^Tc-3PRGD_2_ tumor uptake (T/NT) was observed in the mice treated with Endostar alone or combined with gemcitabine, compared with controls, in agreement with a reduction in tumor growth. On day 14 posttreatment, T/NT in the gemcitabine group was significantly lower than that in the control group. The results of the two-way repeated-measure ANOVA ensure that the treatment was the regimen that resulted in slowing of the growth of the T/NT. Compared with the tumor volume, the difference of the T/NT between treatment groups and the control group was observed earlier. Moreover, the difference of the T/NT in treatment groups including Endostar appeared earlier than that in the gemcitabine group, which may be caused by the earlier mediation of Endostar on neovascularization reduction.

Endostar inhibits neovascular endothelial cells, resulting in reduced integrin expression and decreased accumulation of specific tracers. The reduction in neovascularization may also occur in gemcitabine. The findings were supported by immunofluorescence staining CD31. MVD in all treatment groups was significantly lower than that in the control group. Interestingly, MVD in the gemcitabine group was higher than those in the Endostar group and the combined therapy group, but there was no difference between the latter two groups, which can be explained with the more aggressive neovascularization reduction of Endostar compared to gemcitabine. T/NT was significantly correlated with MVD. ^99m^Tc-3PRGD_2_ SPECT could be a non-invasive method for evaluating MVD.

There were some limitations in the study. The tumor is not very small for the convenience of imaging, so a difference between the treatment groups and the control group occurs late. “Vascular normalization” mechanisms in antiangiogenesis had not been studied in this study, because our research focused on monitoring therapeutic effects with SPECT imaging. The ex vivo biodistribution data of ^99m^Tc-3PRGD_2_ have been investigated in several articles so we focused on the evaluation of imaging quantification.

## Conclusions

Using ^99m^Tc-3PRGD_2_ scintigraphic imaging, the response of antiangiogenesis therapies and chemotherapies can be evaluated in the early stage of treatment, much earlier than the change of tumor volume, providing a new opportunity for individualized treatments and dose optimization.

## Data Availability Statement

The original contributions presented in the study are included in the article/supplementary material. Further inquiries can be directed to the corresponding author.

## Ethics Statement

The animal study was reviewed and approved by the Institute Review Board of Peking Union Medical College Hospital, Chinese Academy of Medical Sciences, and Peking Union Medical College.

## Author Contributions

FL and FW designed the study. YL and CD were responsible for radiosynthesis. KZ and XS were responsible for animal studies. LH helped in the study supervision. The manuscript was drafted by XJ and CD. All authors contributed to the article and approved the submitted version.

## Funding

This work was financially supported, in part, by the National Natural Science Foundation of China (NSFC) projects (81601551, 81801741, 81671722).

## Conflict of Interest

The authors declare that the research was conducted in the absence of any commercial or financial relationships that could be construed as a potential conflict of interest.

## Publisher’s Note

All claims expressed in this article are solely those of the authors and do not necessarily represent those of their affiliated organizations, or those of the publisher, the editors and the reviewers. Any product that may be evaluated in this article, or claim that may be made by its manufacturer, is not guaranteed or endorsed by the publisher.
